# HCV treatment outcome depends on SNPs of IFNL3-Gene polymorphisms (rs12979860) and cirrhotic changes in liver parenchyma

**DOI:** 10.1016/j.heliyon.2023.e21194

**Published:** 2023-10-19

**Authors:** Mohamed Darwish Ahmed Abd Alla, Reham M. Dawood, Hassan Abd EL-Hafeth Rashed, Yasser Mohammed El-Dessouky, Galal AbdElhameed AbuFarrag, Islam Abdelmawla Emran Ammar, Mohamed Mahmoud Abdel-Halim Mahmoud, Ghada M. Salum, Mohamed Zakaria Abu-Amer, Mohamed Abd elrafaa Hassan Sekeen, Mohamed Mousa Ibraheem Heggazy, Ahmed Mohamed Abdulhamid Altanbouly, Mai Abd El-Meguid, Mostafa K. El Awady

**Affiliations:** a Department of Hepatology, Gastroenterology and Infectious Diseases, Faculty of Medicine, Al-Azhar University, Egypt; b Department of Microbial Biotechnology, National Research Centre, Cairo, Egypt

**Keywords:** IFNL3-Gene-SNPs, DAAs, PBMCs-PCR, HCV-Hepatic-changes

## Abstract

The allelic discrimination of IFNL3-(rs12979860 C > T) polymorphism reveals ambiguous associations with the effectiveness of oral HCV treatment. Solitary intra peripheral-blood-mononuclear-cells (PBMCs) HCV-RNA antisense-strands are independently detected in naïve and experienced cases regardless of viremia or hepatic-parenchymal alterations. We examined the frequencies of IFNL3-genetic variants with chronic-HCV-induced liver changes during the sustained virologic response (SVR) by evaluating the PBMCs- HCV-PCR after oral antiviral therapy. **Methods**: Twelve weeks after finishing oral antiviral therapy, the effects of IFNL3-genetic variants were evaluated in three groups of patients: Group-I (n = 25) showed HCV-RNA negativity in both serum and PBMCs-, group II (n = 52) showed positivity of HCV-RNA in PBMCs, and group-III (n = 25) had positive HCV-RNA in serum. The genetic variants of the IFNL3-gene were estimated for all the enrolled cases and correlated with their hepatic image changes. **Results**: IFNL3-(rs12979860) genotyping in post-direct acting antivirals (DAAs) SVR and HCV-relapse revealed: **a)** high frequency of CC-genotype and C-allele in **group I** compared to group **II** (P < 0.005) and group **III**(P ≤ 0.05) when hepatic-parenchyma looks normal by ultrasound **b)** frequent CT-genotype and T-allele in **group II** compared with **I**(P < 0.01) and **III**(P < 0.05) when liver tissues are bright (early cirrhotic-changes) **c)** frequent TT-genotype and T-allele in **group III** relative to **I** (P < 0.05) and **II** (P ≤ 0.08) when liver-tissues appear coarse by ultrasound. **Conclusion**: Outcomes of HCV treatment depend on host IFNL3-gene polymorphism and hepatic-parenchymal changes. A high frequency of wild-CC-genotype and C-allele is observed in patients with normal hepatic parenchyma and that achieved SVR. Solitary relapse in PBMCs occurs on increasing CT-genotype frequency when liver tissues are bright. Serologic relapse is detected when TT-genotype and T-allele are dominant in association with the cirrhotic liver. Therefore, IFNL3-gene-SNP analysis as a genetic predictor in relation to ultra-sonographic hepatic-parenchymal changes could be valuable for selecting the patients with the highest priority for treatment.

## 1 Introduction

The Hepatitis C virus (HCV) causes chronic liver disease in almost 71 million individuals worldwide [1]**.** Egyptians have the greatest rate of HCV genotype-4 infection that is active (10 %), including non-viremic anti-HCV antibody seropositivity and occult-HCV infections [2,3]. Oral direct-acting antivirals (DAAs) [4,5] enhanced HCV clinical outcomes and provided an advantage in analysing host-related factors that may affect HCV-induced liver damage and medication response [6,7].

A newly identified type of persistent HCV infection known as occult hepatitis C virus infection (OCI) has been discovered [8]. Occult HCV infection is characterized by the presence of HCV RNA in the liver and/or the peripheral blood mononuclear cells (PBMCs) in the absence of circulating anti-HCV and HCV RNA [9]. The high hepatitis C incidence (approximate HCV-antibodies’ frequency of 14.7 %) in Egypt may contribute to the high OCI rate [10]. The gold standard to identify OCI with HCV RNA is a hepatic biopsy technique, however recently, the detection of both positive and negative HCV-RNA strands in PBMCs has grown to be utilized as a fallback method that can be employed in case of unavailability of a hepatic biopsy [11].

This article analyzes IFNL3-gene polymorphism in chronic HCV (CHCV) infection with hepatic-parenchymal changes across global populations. The cytokine Interferon-λ3 (IFN-λ3) produced by the interleukin (IL) 28B gene have an antiviral activity and can inhibit HCV replication. In recent years, numerous genome-wide association studies (GWAS) have found a link between liver illness and the two single-nucleotide polymorphisms (SNPs) rs12979860 C/T and rs8099917 T/G on IL28B [12]. A positive correlation was observed between the T-allele of the IFNL3-gene and HCV GT1 cirrhotic patients in the Caucasian population. However, there were inconclusive IFNL3-gene-SNP analyses in a limited number of populations from Asia, Latin America, or the Middle East, who had CHCV-induced hepatic-parenchymal changes [13,14]. Additionally, the genotypes CT and TT of IFNL3-gene were highly reported in CHCV patients with liver cirrhosis, while the CC-genotype was the least common in those with cirrhosis [15]. Conversely, the role of IFNL3-gene-polymorphisms in liver cirrhosis was disputed by other investigators [16,17].

The CC-genotype was reported in the spontaneous clearance of HCV infection. It was addressed as a favorable responder to interferon (IFN) therapy [18,19]. In contrast, others reported an association of the same CC-genotype with a higher frequency of liver cirrhosis and elevated hepatic necro-inflammatory markers [20,21].

The most common method for curing chronic hepatitis patients and preventing HCC development is interferon (IFN) therapy. IFN-λ is one of the multifunctional cytokines involved in regulating the JAK-STAT and MAPK pathways [22].

According to reports, IFN therapy for chronic hepatitis C patients resulted in a biphasic drop in HCV-RNA. Viral kinetics provided insight into antiviral effectiveness. It was proved that HCV-infected patients carrying the IL28B favorable allele showed a rapid decrease in HCV viral replication followed by immune destruction of the HCV-infected cell [23,24]. The association of liver cirrhosis with HCC in CHCV infection is documented. Furthermore, research has approved the relationship between T alleles and/or T SNP of the IFNL3 gene with an increased risk of liver cirrhosis and subsequently with a higher probability of developing HCC [25].

In 2019, a relationship was shown between between IFNL3-gene TT-genotype and HCV post-DAAs-treatment relapse/resistance and between wild-type CC-genotype of IFNL3-gene and sustained-virologic-response (SVR) [26]. In the same year, other researchers found that post-DAAs-SVR was more common in wild-C than the T-allele of IFNL3-gene [27].

The high cure rates reported with DAAs in many studies were based mostly on viral clearance from serum rather than PBMCs. Contrarily, other investigators reported that more than 60 % of the studied populations had positive HCV-RNA in PBMCs at the end of the 12th week after treatment of CHCV infection with DAAs [28,29]. The disparity between serum and PBMCs-PCRs in calculating SVR after DAAs-therapy should promote continued IFNL3-gene analysis, despite previous discrepancies in its association with treatment outcomes [30].

The current research aims to shed light on IFNL3-(rs12979860) genotyping variations in response to DAAs-treatment in the following situations: a) SVR with negative HCV-RNA in serum and PBMCs; b) solitary intra-PBMCs HCV infection with negative serum HCV-PCR; c) positive serum HCV-RNA. In addition, the IFNL3-(rs12979860) polymorphism associated with DAAs treatment outcomes was correlated with ultrasound (US) grading of CHCV-induced hepatic-parenchymal changes.

## 2 Subjects and methods

### 2.1 Subjects

**Fig. 1:**
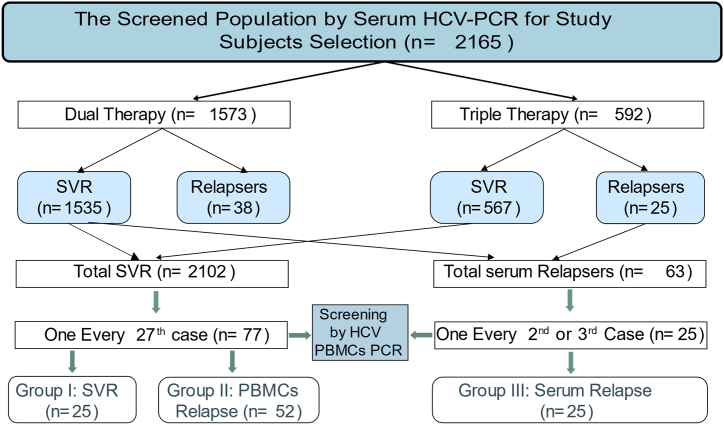
Grouping of the randomly selected study populations at the end of the 12th week after completing a full DAAs treatment course. Subjects in group I had negative serum and peripheral blood mononuclear cells (PBMCs) PCRs: those in group II showed positive solitary intra-PBMCs HCV-PCR with negative serum viral PCR: cases in group III presented with positive serum HCV-RNA by PCR. Dual therapy = SOF + DAC; Triple therapy = SOF + DAC + RBV. SVR= Sustained Virologic Response.

Patients were selected from the Al-Azhar University Specialised Hospital's outpatient clinic for hepatology, gastroenterology, and infectious diseases. All the recruited patients had HCV genotype 4a. Three months after the end of treatment (EOT) with DAAs, 102 cases were randomly selected, as demonstrated below in Fig. 1. The studied groups were matched regarding age, gender, and treatment regimen. The age range of the selected subjects was between 18 and 75 years. The exclusion criteria included co-infection with HIV or HBV, pregnant female, hepatocellular carcinoma, total serum bilirubin more than 3 mg/dl, INR more than 1.7, serum albumin less than 2.8 g/dl and platelet count less than 50,000/mm.

Both serum and PBMCs HCV -PCR screened the 102 selected subjects. Then the subjects were divided into three groups: I (n = 25) showed negative HCV-RNA in both PBMCs and serum, II (n = 52) with negative serum HCV RNA but positive HCV-RNA in PBMCs, and III (n = 25) with still positive HCV-RNA in serum. All study subjects were treated with DAAs (400 mg SOF+60 mg DCV) ± RBV (up to 1200 mg according to body weight) for three months in accordance with Egyptian national guidelines for HCV therapy [31]. The current study has been approved by the Institutional Ethical Review Board (Research Ethics Committee of Al Azhar University, IRB, 0000013.) according to the Declaration of Helsinki 1975, revised in 2008, and carried out with the knowledge and consent of the human subjects. Written informed consent was obtained from all the enrolled participants.

### 2.2 Detection of serum HCV-RNA by real-time PCR

The QIAamp Viral RNA Mini Kit (Qiagen, Milan, Italy) was used to extract the HCV-RNA from serum according to standard manufacturer's instructions, then the extracted viral RNA was amplified by real-time PCR by using Artus HCV QS RGQ Kit (Qiagen) following the manufacturer's protocols. The cycling conditions of amplification was initial incubation for 30 min at 51 °C, a second step of 10 min at 95 °C, followed by 50 cycles of 30 s at 95 °C and 1 min at 60 °C, followed by 40 cycles at 95 °C for 15 s, 60 °C for 1 min and 72 °C for 30 s. The amplified Fluorescence signal was detected at annealing/extension step of each cycle. Rotor-Gene real-time PCR machine (Qiagen, Santa Clarita, CA) was used for performing the amplification.

### 2.3 Detection of PBMCs- HCV-RNA by PCR

The single-step method [32] was used to isolate the total cellular-RNA from the PBMCs that was modified later [33,34], and then the PBMCs- HCV-RNA PCR was performed as described in Ref. [33].

### 2.4 Extraction of DNA

Genomic DNA was extracted from 200 μl of whole blood by Qiagen DNA extraction kit (Qiagen, Milan Italy) following the manufacturer's protocols. The extracted DNA was evaluated for purity and quantity by using NanoDrop (USA; UV-VIS-Spectrophotometer Q 5000).

### 2.5 IFNL3-gene-polymorphism screening

The IFNL3 polymorphism (rs12979860 C > T) was detected using an allelic discrimination real-time-PCR protocol based on the pre-validated TaqMan-MGB™ probe (Applied Biosystems, Foster City, California, US). Briefly, 1.25 μL of a 40X-combined-primer and probe-mix (ABI/Life Technologies, USA) was incorporated to 12.5 μL of 2X TaqMan® Universal-PCR-master-mix (ABI/Life-Technologies, USA) and DNA template and then complete to 25 μL final volume with DNase/RNase-free-water (Invitrogen/Life-Technologies, USA). The thermal profile of amplification was 95 °C for 10 min, 95 °C for 15s, and 60 °C for 1 min. The final two steps were performed 40 times. The Rotor-Gene real-time PCR machine (Qiagen, Santa Clarita, CA) was used for the PCR run. The Statistical Package for The Social Sciences (SPSS version 16.0; SPSS, Chicago, IL) was used to create Allelic discrimination plots.

### 2.6 Assessment of hepatic-parenchymal alterations (early fibrosis versus late cirrhosis)

In all enrolled patients, CHCV-induced liver tissue alterations were assessed using US images and linked with the FIB4-scoring system. In the current research, the radiologist on duty in the interventional ultrasound suite had at least five years of experience performing abdominal sonography and applied real-time ultrasound using a 3.5–5.0 MHz convex transducer. Ultrasounds of liver lobes and spleen were performed, and a combined impression was derived. The elaborated description of cirrhotic findings on US examination included: a) surface nodularity (88 % sensitive, 82–95 % specific); b) overall coarse and heterogeneous echotexture; c) segmental hypertrophy/atrophy; d) caudate width to right lobe width >0.65 (43–84 % sensitive, 100 % specific); e) reduction of the transverse diameter (<30 mm) of the medial segment of the hepatic left-lobe (segment). Portal hypertension was addressed by detecting the enlarged portal vein: >13 mm (42 % sensitive, 95–100 % specific).

### 2.7 Statistics

SPSS 16.0 (IBM; NY, USA) analyzed the current data set. Results were compared with the X2 or Fisher exact tests; continuous variables were expressed in terms of means and standard deviations. Ordinal and nominal categorical results were addressed as numbers and percentages. “Epi info CDC software was used in statistical analysis of the current study data” [35]. P < 0.05 indicated a significant difference.

## 3 Results

### 3.1 The demographic data of the studied groups

The established research protocol helped to limit variations in demographic and clinical data, as cases were selected according to the national guidelines for chronic HCV management. However, Alpha-fetoprotein showed a significant difference among groups (P = 0.03). No significant difference was observed in age and gender between the three groups (P = 0.44 for age and P = 0.7 for gender) as illustrated in Table 1, despite the predominance of male-gender within each group when compared to female (P < 0.001).

**Table 1:** Demographic features of the studied groups.

	Group I N = 25	Group II N = 52	Group III N = 25	P value
Dual treatment	10*	36*	14	<0.001 , Ii vs iii
Triple treatment	15	16	11	0.3
Age	49 ± 9	51.8 ± 8	51	0.44
Sex(Male/Female)	24/1	50/2	23/2	0.7
^1^Bilirubin Total (mg/dl)	0.94 ± 0.94 a	0.91 ± 0.74	0.94 ± 0.41	0.99
^1^AST (U/L)	35.8 ± 26.4	40.2 ± 20.6	41.1 ± 22.8	0.66
^1^ALT (U/L)	41.32 ± 23.44	40.6 ± 22.8	41.7 ± 27	0.98
^1^INR	1.04 ± 0.06	1.09 ± 0.12	1.09 ± 0.12	0.13
Albumin (g/dl)	4.3 ± 0.6	4.37 ± 0.45	4.15 ± 0.43	0.2
WBCs( × 10^3^/cmm^3^)	6.6 ± 2.6	6.7 ± 2.5	6.1 ± 2.1	0.7
^1^PLT ( × 10^3^ cmm^3^)	209.5 ± 75	205.3 ± 55	199 ± 116	0.9
^1^AFP (ng/mL)	6.9 ± 6.3	8.3 ± 14	14.6 ± 14.1	0.03
Creatinine (mg/dl)	0,87 ± 0.2	0.9 ± 0.3	0.8 ± 0.3	0.7
HCV RNA PCR ( × 10^3^ IU/ml)	4.8 ± 3.7	5.1 ± 3.3	4.9 ± 3.2	0.96

1 AST points to serum aspartate aminotransferase; ALT points to serum alanine aminotransferase, INR points to the international normalized ratio of prothrombin time; PLT points to platelets count; AFP points to alpha-fetoprotein; p-value <0.01 upon comparing group II &I.

### 3.2 Relationship between DAAs therapeutic regimen and relapse category in the studied groups

The distribution of both dual (SOF and DCV) and triple (SOF and DCV plus RBV) regimens in the studied groups (n = 102) was illustrated as the following: Subjects who received dual regimen (n = 60) were respectively presented in groups I, II, and III as 16.67 % (n = 10), 60 % (n = 36) and 23.33 % (n = 14). The received triple-regimen by 42 patients was administered in 35.71 % (n = 15), 38.09 % (n = 16), and 26.19 % (n = 11) of groups I, II, and III, respectively. Analysis of the results above revealed that Group II had a higher intake of dual therapy than Group I and Group III (P 0.001 and P 0.001, respectively). At the same time, triple regimen administration had the same rates in the three groups (P > 0.3). There was increased triple-regimen intake in Group I (P = 0.03) and dual regimen administration in Group II (P = 0.04). In contrast, Group III showed an insignificant difference between the two regimens. Accordingly, dual therapy seems to be strongly associated with an increased frequency of cellular relapses.

### 3.3 Detection of the antisense HCV-RNA-strands in PBMCs in relapsers (groups II and III)

The profiling of the sense and antisense RNA strands within PBMCs in the relapsers after DAAs-therapy in groups II and III was described as the following: Groups II and III showed intra-PBMCs HCV-RNA sense and antisense strands in 41 out of 52 (78 %) and 14 out of 25 (56 %), respectively. In comparison, solitary antisense RNA strands were detected in 4 out of 52 (7.69 %) and 5 out of 25 (20 %) in groups II and III, respectively. A total of 45 out of 52 relapsers in Group II and 19 out of 25 in Group III had actively replicating intracellular viral genome. While HCV-RNA sense-strands were only observed in 7 out of 52 (13.46 %) and 6 out of 25 (24 %) of the remaining relapsers in groups II and III, respectively.Data revealed that the presence of HCV-RNA antisense strand reflected the active viral replication in Group II (P < 0.001) and Group III (P < 0.005).

### 3.4 Correlation of IFNL3-genetic variants with US hepatic-parenchymal changes

The recessive genetic model has been used. The correlation of IFNL3 polymorphism (rs12979860 C > T) with US liver changes in all study groups was illustrated in Table 2. Variations of IL-28B-gene-SNPs in **normal-hepatic-US appearance** indicated the following: **a)** higher frequency of wild-type CC in post-DAAs treatment in group I compared to Group II (P = 0.004) and Group III (P = 0.05); **b)** CT-genotype was seen more often during solitary intra-PBMCs HCV viral infection in Group II and SVR subjects (Group I) compared with serum HCV-RNA in Group III (P < 0.001and 0.02, respectively), with non-significant change when comparing groups, I with II (p = 0.38); **c)** non-significant difference in TT-genotype was demonstrated on comparing the three groups with each other (P > 0.4). **In bright liver (early-parenchymal-changes) by ultrasound**, the obtained data in Table 3 revealed that: **a)** CT-genotype was seen more often in solely intracellular HCV-infection (Group II) than in SVR subjects (Group I) (P = 0.007) and Group III patients who retained detectable serum-RNA by PCR (P = 0.03); **b)** wild-type CC and TT-genotypes were almost equally distributed in the three groups. **In patients with coarse-liver by ultrasound,** current findings indicated the following: **a)** TT-genotype was seen more often in Group III than in Group I (P = 0.04) and Group II (P = 0.17); **b)** non-significant differences were observed in wild-type CC and CT-genotypes when comparing the three groups with each other (P > 0.1).

**Table 2:** The IFNL3 polymorphism (rs12979860 C > T) gene in relation to hepatic ultrasound (US) image variations.

Study groups	Normal liver by the US (n = 31)			Bright liver by the US (n = 55)			Cirrhotic liver by the US (n = 16)		
	CC	CT	TT	CC	CT	TT	CC	CT	TT
Group I	8 (26 %)	6 (19.3 %)	1 (3.2 %)	0 (0 %)	5 (9 %)	5 (9 %)	0 (0 %)	0 (0 %)	0 (0 %)
Group II	4 (139 %)	10 (32 %)	2 (6.4 %)	4 (7.2 %)	17 (31 %)	9 (16.3 %)	4 (25 %)	1 (6.2 %)	1 (6.2 %)
Group III	0 (0 %)	0 (0 %)	0 (0 %)	5 (9 %)	7 (12.7 %)	3 (5.4 %)	1 (6.2 %)	4 (25 %)	5 (31 %)
P Value: I vs II I vs III II vs III	0.11 **0.004** 0.05	0.38 **0.02** **<0.001**	1 1 0.49	0.11 0.05 1	**0.007** 0.76 **0.03**	0.39 0.71 0.12	0.10 1 0.33	1 0.33 0.10	1 **0.04** 0.17

**Table 3:** Correlation of IFNL3 (rs12979860) alleles (T or C) with grads of hepatic parenchyma by Ultrasound (US) in post-DAAs therapy outcomes.

Study groups	Normal liver by the US (n = 62)		Bright liver by US (n = 110)		Coarse liver by the US (n = 32)	
	C allele	T allele	C allele	T allele	C allele	T allele
Group I	22 (35.4 %)	8 (12.9 %)	5 (4.5 %)	15 (13.6 %)	0 (0 %)	0 (0 %)
Group II	18 (29 %)	14 (22.5 %)	25 (22.7 %)	35 (31.8 %)	9 (28.1 %)	3 (9.3 %)
Group III	0 (0 %)	0 (0 %)	17 (15.4 %)	13 (11.8 %)	6 (18.7 %)	14 (43.7 %)
P Value: I vs II I vs III II vs III	0.56 **<0.001** **<0.001**	0.23 **0.006** **<0.001**	**<0.001** **0.01** 0.22	**0.002** 0.84 **<0.001**	**0.002** 0.**02** 0.55	0.23 **<0.001** **0.003**

On comparing the sum of IL-28B-gene-SNP variation in all study groups within each US category regardless of infection status, the following findings were noted: **A) In normal US hepatic appearance**: both wild-type CC (12/31) and CT genotype (16/31) were more frequent than TT-genotype (3/31) (P = 0.016, and 0.007, respectively), with an insignificant difference when comparing the wild-type CC to the CT-genotype (P = 0.44). **B) In bright hepatic-parenchymal-tissues by the US**: The CT-genotype (29/55) was seen more often than wild-type-CC (9/55) and TT-genotype (17/55) (P = 0.001, and 0.03, respectively); with no difference on comparing the wild-type CC with TT-genotype (P = 0.11). **C) In Coarse liver parenchyma by the US**: Frequencies of the wild-type CC (5/16), CT (5/16), and TT (6/16) genotypes showed insignificant differences.

Regardless of post-treatment virologic status, the distribution of CT-genotype in chronic HCV-infected patients (50/102) was significantly higher than the wild-type CC (26/102) and the mutated TT genotype (26/102) (P < 0.001). This finding reflected the following **a.** an increased susceptibility to develop chronic HCV infection, **b.** an increased resistance to DAAs therapy, **c.** higher probability of persistent HCV RNA in PBMCs, in CT genotype (n = 29) when compared to serologic relapses (n = 5) and SVR (n = 16) among a total of 50 intracellular HCV infections, P < 0.05.

In patients with normal hepatic ultrasound: a) Group I had a greater frequency of wild-type CC than Group II (P = 0.004) and Group III (P = 0.05); b)Individuals in Group II exhibiting only intracellular HCV infection had the CT-genotype more frequently than those in Group III who had RNA-seropositive individuals (P 0.001).In patients with bright liver by ultrasound: a) (Group II) in solitary intracellular Infection had CT-genotype more frequent than Group I in SVR subjects (P = 0.007) or those in Group III with positive serum RNA by PCR (P = 0.03); b) In the three groups, the CC and TT wild-type genotypes were approximately equally distributed. In cases with coarse liver by US image: **a)** Group III (positive serum HCV-PCR) had a higher prevalence of the TT-genotype in comparison to Group I (P = 0.04) and Group II (P = 0.17); **b)** no significant difference was observed in both wild-type CC and CT-genotype on comparing the three groups to each other. The bold indicates a significant value (P-value ≤0.05 is considered statistically significant). Fisher's exact 2-tailed P was used in data analysis.

**Fig. 2:**
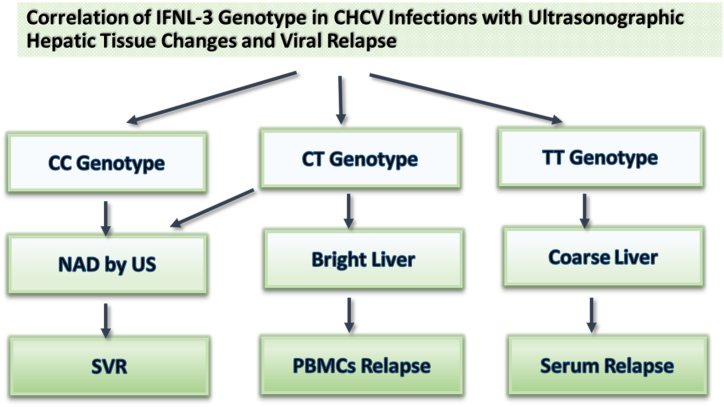
Relationship of IFNL3 polymorphism (rs12979860 C > T) with viral relapse in various grades of hepatic parenchymal changes after treating CHCV infection with DAAs. The wild CC nucleotides were dominant in the SVR when hepatic tissues looked normal by the ultrasound (US). At the same time, the CT genotype was associated with peripheral blood mononuclear cells (PBMCs) relapse when hepatic parenchyma looked normal or bright (fibrotic = early changes by the US). TT genotype was more frequent in serum relapse when liver tissues were cirrhotic. **NAD**, no abnormality detected; **US**, ultrasound; **SVR**, sustained virologic response, **CHCV**, Chronic HCV.

Fig. 2 summarized frequencies of the wild-type CC, CT, and TT-genotypes, respectively, in SVR, solitary PBMCs, and serum HCV relapses in relation to hepatic parenchymal changes after DAAs-therapy. Data presented in the figure concluded that the wild-type CC was associated with a full response to DAAs therapy when we did not detect changes in liver tissues by US. The CT-genotype was recognized in cases with HCV-PBMCs relapse predominantly in those who developed bright liver image (early stages of liver cirrhosis) by the US. The mutant TT-genotype was mainly observed with serologic HCV-relapse, particularly in those who presented with liver cirrhosis.

### 3.5 Correlation of IFNL3-allele frequencies with the evaluated liver tissue changes by ultrasound (US)

The relationship of C or T-allele frequency with various US images of hepatic parenchyma in the studied CHCV cohort was illustrated in Table 3. When **no abnormality detected** (NAD) by US-image (n = 62 allele) was addressed, the C-allele of IL28-gene was recognized more often in both SVR (group I) and cellular relapse (group II) compared to serologic relapse in group III (P < 0.001). In contrast, the T-allele was frequently recognized more in group II compared with group I (P = 0.006) and III (P < 0.001). When liver tissues appear **bright in early hepatic-parenchymal changes** by US (n = 110 allele), the T-allele was more recognizable in group II than SVR patients in group I (P = 0.002) and HCV-RNA seropositive patients in group III (P = 0.005). In **coarse hepatic-parenchyma** by the US (n = 32-allele), the T-allele was more frequently seen in group III compared to SVR in group I (SVR) (P < 0.001) and cellular relapses in group II (P < 0.001). On the other hand, the C-allele was recognized more often in cellular relapse when liver parenchyma was bright (P = 0.001) or cirrhotic (P = 0.002) compared to sustained virologic responders who had bright and cirrhotic liver, respectively.

Comparing the frequency of C or T-allele within each group with correlation to US image categories revealed the following: **a)** the SVR group had a significantly higher frequency of C-allele when US showed no abnormality (22/62) compared to bright (5/110) (P < 0.001) or cirrhotic liver tissues (0/32) (P < 0.001), **b)** cellular relapse in group II had more T-allele in the bright liver (35/110) compared to normal-appearing US image (14/62) (P = 0.22), and coarse liver (3/32) (P = 0.01), **c)** serologic relapse in group III showed a significantly higher frequency of T-allele in cirrhotic (14/32) compared to normal-appearing (0/62) (P < 0.001), and bright hepatic parenchyma (13/110) (P = 0.002). Fisher's exact 2-tailed P was used to analyze data.

In normal-appearing liver tissues by the US, the C allele was more frequent in groups I and II than in Group III (P < 0.001). In contrast, the T allele was significantly recognized in Group II compared to Group I (P = 0.006) and III (P < 0.001). In bright liver tissue (early hepatic parenchymal changes by image, the T allele was associated with intracellular HCV infection (group II) when compared to SVR in Group I (P = 0.002) and serologic viral relapse in Group III (P < 0.001). In cirrhotic liver by sonographic examination, the T allele was more frequent in HCV RNA seroconversion (Group III) compared to SVR in Group I (P < 0.001) and cellular relapse in Group II (P < 0.001). The C allele showed a higher association with cellular HCV relapse in bright (P = 0.001) and cirrhotic (P = 0.002) liver, respectively, compared to SVR in bright and cirrhotic liver parenchymal changes. The bold indicates a significant value (P-value ≤0.05 is considered statistically significant). Fisher's exact 2-tailed P was used in data analysis.

**Fig. 3:**
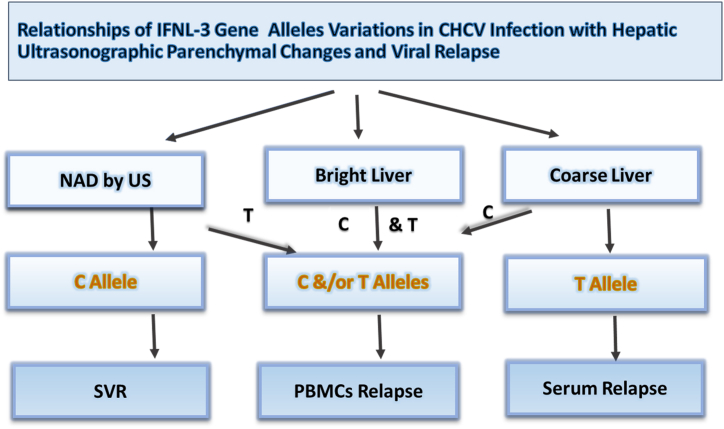
Distribution of IFNL3 (rs12979860) gene C and T alleles in sustained virologic response (SVR) and HCV relapse correlates to grades of hepatic parenchymal changes by ultrasound (US). When hepatic US concluded no abnormality detected (NAD), the C allele was dominant in SVR, while the T allele was associated with PBMC-HCV relapse. In bright hepatic parenchyma by US, both C and T alleles were predominantly associated with PBMCs HCV relapse. While in coarse liver tissues by the US, there was predominant recognition of the T allele in HCV serum relapse and frequent detection of the C allele in persistent HCV-RNA in PBMCs (cellular relapse vs. non-response). **CHCV**, chronic HCV; **US**, ultrasound; **NAD**, no abnormality detected; **SVR**, sustained virologic response.

The distribution of C and T-alleles of IFNL3-(rs12979860) was evaluated in the recruited three post-DAAs-therapy groups, as described in Fig. 3. The studied group was classified according to the ultra-sonographic appearance of hepatic tissues into three categories: a) NAD, b) early hepatic parenchymal changes defined as bright liver, and c) coarse liver surface defined as hepatic cirrhosis. Fig. 3 showed that: a) SVR and PBMCs-relapse were respectively associated with C and T alleles when hepatic US had NAD category b) PBMCs relapse was associated with C or T allele when liver US showed bright category during early hepatic parenchymal changes c) serum and PBMCs HCV relapses were respectively associated with T and C alleles when the hepatic US had the coarse category.

## 4 Discussion

The IFNL3-gene is found with IL28A and IL29 in a cytokine cluster at 19q13.13 and codes for IFNλ3. The upstream haplotype associated with viral clearance at SNPs rs12979860 and rs8099917 is CT, and the persistence haplotype at these SNPs is TG [36]. The CC genotype of IFNL3-(rs12979860) is associated with distinct post-Peg-IFN treatment outcomes [18,21]. The introduction of DAAs in 2014 raised more debate regarding effectiveness because of the variation in therapeutic outcomes. The role of IFNL3-gene polymorphism in DAAs treatment outcomes remains under investigation due to worldwide variations in viral relapse/breakthrough rates [[26], [27], [28]]. Several previous studies reported the correlations of IFN-λ polymorphism with treatment response are summarized in Table 4.

**Table 4:** Different studies for the correlation of IFN-λ polymorphism with clinical outcomes of hepatitis C.

	Author	population	Sample size	Journal	The conclusion
1	Tanaka et al., 2009	Japanese	N/A	Nature Genetics	Individuals with the T allele displayed decreased levels of IFN- expression in peripheral blood mononuclear cells via real-time quantitative PCR experiments [45].
2	Tillman et al., 2011	United States	325 Patients	Journal of Hepatology	IFN- λ CC genotype is associated with lessened pronounced disturbances of lipid metabolism and a lower incidence of steatosis in HCV patients [46].
3	Gao et al., 2012	United States	N/A	Journal of Hepatology	An interesting review article concluded that STAT1 in hepatocytes is predominantly activated by IFN-λ. Upon STAT1 activation in liver non-parenchymal cells, it Inhibits liver fibrosis and promotes the inflammatory response. On the other side, a higher level of IFN-λ expression was documented in patients with the CC genotype. The authors recommended the IFN-λ treatment as it is less likely to induce the hematopoietic and neurological side effects documented with IFN-α therapy usage [47].
4	Langhans et al., 2011	Caucasian	134 Patients	Journal of Hepatology	Carriers of the rs12979860 C allele associated with the resolution of HCV infection exhibited about two folds increments of IFN-λ levels [48].
5	Salum et al., 2020	Egypt	495 Patients	Genes & Diseases	Sofosbovir/Daclatasvir combination enhanced the sustained virological response rates in HCV genotype 4a infected patients, with the best rates in those lacking the T allele [27].
6	de Souza et al., 2018	Brazil, genotype 1	125 Patients	BioMed Research International	The SNPs rs12979860 and rs8099917 on IFN-λ are related to IFN treatment success [49].
7	Hiromi et al., 2010	Asian, American, and European population	364 Patients	Journal of Hepatology	In Japanese patients with HCV genotype 1b infection, this study found genetic diversity near the IL28B gene and aa replacement of the core region as predictors of sustained virological response to a triple therapy of telaprevir/PEG-IFN/ribavirin. These findings may contribute to our understanding of the progression of liver diseases and help to guide treatment strategies for patients with chronic HCV infection [50].
8	ValliDeRe et al., 2014	Caucasian patients	1050 patients with chronic infection+ 3882 Caucasian patients	BioMed Research International	Their data verified the relation between IFN-λ C allele and HCV spontaneous clearance in more than three thousand patients [51].
9	Bota et al., 2013	SYSTEMATIC REVIEW	1641 Patients	Clin Drug Investig	This review elucidated the efficacy of the IFN-λ polymorphism on the SVR rate in HCV chronic patients regardless of their therapeutic status [52].

Infection with CHCV causes liver disease that can vary from hepatic stiffness and fibrosis to cirrhosis [37,38]. CHCV infection leads to the development of cirrhotic liver in only a small minority of the infected population, illustrating whether particular host elements are at play. Screening for HCV-cellular relapse by PBMCs-HCV-PCR suggests incomplete evaluation of SVR by serum HCV-PCR alone [28,29]. Therefore, further research is needed to examine the link between gene polymorphism and the incidence of various degrees of hepatic parenchymal alterations before proposing the anti-viral therapeutic regimens. The current research used a third group of patients who had solitary positive HCV-RNA in PBMCs at the end of the 12th week after EOT, with antisense and/or sense RNA strands as a sign of active viral replication, given the definition of cellular relapses as documented previously by other investigators [28,29].

HCV RNA detection in serum determines post-DAA response, SVR, no response, or relapse. SVR is assessed at the end of the 12th week after the end of treatment (EOT) when serum HCV-PCR turns negative [4,5,39]. When adding PBMCs-PCR to HCV diagnostic protocol, other researchers suggested different definitions of nonresponse, relapse, and SVR, given the persistent detection of intracellular HCV-RNA genomic strands [32,40]. According to reports, monitoring of patients of both naïve and experienced with solitary positive HCV in PBMCs infection was linked to viral-RNA seroconversion [3]. Subjects presented with SVR had negative serum and PBMCs HCV by PCR, while those with serologic relapse had positive serum viral-RNA-PCR after 12 weeks from EOT [3,41].

Abdominal ultrasound (US) screening results show limited variation because of differences among experienced operators (hand bias) during solid organ evaluation. In the current research, the US screening of liver tissues was performed using a real-time 3.5–5.0 MHz convex transducer. The radiologists had at least five years of experience performing sonography in the intervention radiology suite. So, hand bias would have a minimum influence on generating the same data sets by different radiologists with long-term expertise. Although liver tissue sonography may not be enough to evaluate hepatic parenchymal changes, the US would be conclusive in addressing the following clinical presentations: **a)** no abnormality detected when liver tissues appear normal; **b)** cirrhotic liver by recognizing coarse hepatic surface. The third clinical category is a bright liver that requires differentiation between early CHCV-induced hepatic parenchymal changes and other liver pathology (e.g., fatty liver). In our research, almost all patients presented with bright liver were normotensive and nondiabetic. They had average BMI, normal liver enzymes and lipid profile, positive anti-HCV IgG antibody serology, and positive serum HCV-RNA-PCR. The above-mentioned US criticism (hand bias) was managed by adding a FIB 4 scoring system compared to hepatic imaging by the US to evaluate their compatibility and validate US use in the current research as demonstrated previously [28].

Evaluating the HCV-RNA in PBMCs significantly altered the conclusions obtained from the other studies regarding whether IFNL3-genetic variants exhibited either positive [13,14,42] or negative [43] correlations with anti-HCV DAAs treatment outcomes. The association between IFNL3 genotyping with PBMCs HCV relapse in different grades of liver parenchymal changes is addressed for the first time in the current study. Cellular relapse frequently occurs in bright liver by US (i.e., early cirrhotic changes) regardless of IFNL3-gene makeup. However, the presence of the C-allele seems to ameliorate CHCV infection and improve DAAs therapy outcomes. At this point, we can then conclude that: **a)** dominance of the C-allele in normal liver parenchyma by the US is associated with clearance of both serum and cellular HCV infection (SVR); **b)** recognition of C-allele dominance in bright (early fibrotic) or coarse (cirrhotic) liver leads to clearance of viremia despite the persistence of PBMCs HCV-RNA. Contrarily, the dominance of the T-allele seems to be an unfavorable association as it leads to worse HCV infection outcomes and an increase in viral relapse after DAAs therapy. Therefore, the other facet of the picture will be **a)** dominant T-allele is associated with HCV serologic relapse in the cirrhotic liver with the coarse surface by the US; **b)** frequent recognition of T-allele in normal and bright hepatic tissues by the US is accompanied by persistent intra-PBMCs HCV-infection despite serum viral-RNA clearance.

Pretreatment submission of CHCV-patients to IFNL3-(rs12979860) typing in relation to hepatic parenchymal changes elaborates one of the following clinically applied outcomes: **a**. **SVR** in wild CC-genotype when liver tissues look normal by the US, which may favor the feasibility of reducing the duration of the anti-viral treatment course; **b**. **Solitary intra-PBMCs persistence of HCV-RNA** in wild CC and CT-heterozygotes when liver tissues are fibrotic (bright liver by the US), a situation that may require an extension of DAAs course duration beyond the scheduled termination point until the elimination of intracellular viral-RNA; **c**. **Serologic viral relapse** (positive serum HCV-PCR) in TT-genotype when liver tissues are cirrhotic (coarse hepatic surface by the US), a clinical presentation that requires either viral genome screening for resistance-associated variant (RAV) or adding Ribavirin to the currently used regimen or using different anti-viral regimens for a maybe longer duration than the anticipated endpoint of treatment. In addition, the predominance of IFNL3-gene C or T-allele in CHCV infection reveals one of the following situations: **d**. **SVR and PBMCs-relapse** are respectively seen in normal and cirrhotic liver tissues when the C-allele is dominant; **e**. **PBMCs and serologic relapses** are more frequent in normal (NAD by the US) and cirrhotic hepatic parenchyma (coarse liver surface by the US) on the dominance of the T-allele; **f**. **Cellular relapse** is found more often in bright liver tissues by US (early hepatic parenchymal changes) with the dominance of either C or T-alleles. Further studies are needed to address the relationship between the IFNL3-gene SNPs and DAAs regimens and new anti-viral therapies, other than the currently used therapeutic regimens, which have been described in other HCV management protocols [39,44], and drug administration for either extended or shortened durations as compared to current or previous studies [3]. The current study's limitations are as follows: The histopathological changes are not determined by liver biopsy. We should also measure the IFNL3 levels and correlate their expression level with genotyping status. Finally, different genetic variants within the same gene should be investigated to elucidate the full association of this gene with the clinical outcome.

## 5 Conclusion

IFNL3-(rs12979860) polymorphism strongly correlates with treatment outcomes in various virally induced hepatic-parenchymal changes. SVR cases are more likely to have the genotype CC and higher incidence of C allele, particularly in normal hepatic parenchyma by ultrasound (US). The heterozygous IFNL3 CT-genotype has been identified in solitary intra-PBMCs HCV relapse cases with early fibrotic liver parenchyma (bright liver by the US). T and C alleles are associated with solitary PBMC relapse in normal and cirrhotic liver. Finally, the TT-genotype and, subsequently, T-allele frequency is dominant in HCV-serologic relapse in association with liver cirrhosis (coarse surface by the US). Pre-treatment screening of the IFNL3-gene polymorphism (rs12979860) for large-scale studies is needed to further confirm these findings in different therapeutic regimens and various grades of HCV-induced liver tissue changes.

## Data availability statement

Data included in article/supplementary material/referenced in article.

## Author Agreement/Declaration

All authors reviewed and approved the final form of the submitted paper was viewed and accepted by all authors. The authors declare that this work is original and that all information, including all tables and figures, was created by the authors and has not been published or is currently under consideration elsewhere.

## CRediT authorship contribution statement

**Mohamed Darwish Ahmed Abd Alla:** Writing – original draft, Visualization, Supervision, Project administration, Investigation, Formal analysis, Data curation, Conceptualization. **Reham M. Dawood:** Writing – review & editing, Methodology. **Hassan Abd EL-Hafeth Rashed:** Writing – review & editing. **Yasser Mohammed El-Dessouky:** Writing – review & editing. **Galal AbdElhameed AbuFarrag:** Data curation. **Islam Abdelmawla Emran Ammar:** Resources, Data curation. **Mohamed Mahmoud Abdel-Halim Mahmoud:** Resources. **Ghada M. Salum:** Validation, Methodology. **Mohamed Zakaria Abu-Amer:** Resources. **Mohamed Abd elrafaa Hassan Sekeen:** Resources. **Mohamed Mousa Ibraheem Heggazy:** Resources. **Ahmed Mohamed Abdulhamid Altanbouly:** Resources, Formal analysis, Data curation. **Mai Abd El-Meguid:** Validation, Methodology, Formal analysis. **Mostafa K. El Awady:** Writing – original draft, Supervision.

## Declaration of competing interest

The authors declare that they have no known competing financial interests or personal relationships that could have appeared to influence the work reported in this paper.
